# Roles of Chitosan as Bio-Fillers in Radiation-Vulcanized Natural Rubber Latex and Hybrid Radiation and Peroxide-Vulcanized Natural Rubber Latex: Physical/Mechanical Properties under Thermal Aging and Biodegradability

**DOI:** 10.3390/polym13223940

**Published:** 2021-11-15

**Authors:** Arkarapol Thumwong, Worawat Poltabtim, Patcharaporn Kerdsang, Kiadtisak Saenboonruang

**Affiliations:** 1 Department of Applied Radiation and Isotopes, Faculty of Science, Kasetsart University, Bangkok 10900, Thailand; arkarapol.th@ku.th (A.T.); wp.worawat@gmail.com (W.P.); patcharaporn.ker@ku.th (P.K.); 2 Department of Materials Science, Faculty of Science, Kasetsart University, Bangkok 10900, Thailand; 3 Specialized Center of Rubber and Polymer Materials in Agriculture and Industry (RPM), Faculty of Science, Kasetsart University, Bangkok 10900, Thailand; 4 Special Research Unit of Radiation Technology for Advanced Materials, Faculty of Science, Kasetsart University, Bangkok 10900, Thailand

**Keywords:** chitosan, radiation, peroxide, natural rubber latex, biodegradability, mechanical properties, thermal aging, vulcanization

## Abstract

Although natural rubber was regarded as biodegradable, the degradation is a time-consuming process that could take weeks or months for any degradation or substantial weight loss to be observable, resulting in the need for novel processes/methods to accelerate the rubber degradation. As a result, this work investigated the potential utilization of chitosan (CS) as a biodegradation enhancer for radiation-vulcanized natural rubber latex (R-VNRL) and hybrid radiation and peroxide-vulcanized natural rubber latex (RP-VNRL) composites, with varying CS contents (0, 2, 4, or 6 phr). The R-VNRL samples were prepared using 15 kGy gamma irradiation, while the RP-VNRL samples were prepared using a combination of 0.1 phr tert-butyl hydroperoxide (*t*-BHPO) and 10 kGy gamma irradiation. The properties investigated were biodegradability in the soil and the morphological, chemical, mechanical, and physical properties, both before and after undergoing thermal aging. The results indicated that the biodegradability of both the R-VNRL and RP-VNRL composites was enhanced with the addition of CS, as evidenced by increases in the percentage weight loss (% weight loss) after being buried in soil for 8 weeks from 6.5 ± 0.1% and 6.4 ± 0.1% in a pristine R-VNRL and RP-VNRL samples, respectively, to 10.5 ± 0.1% and 10.2 ± 0.1% in 6-pph CS/R-VNRL and 6-pph CS/RP-VNRL composites, respectively, indicating the biodegradation enhancement of approximately 60%. In addition, the results revealed that the addition of CS could increase the value of tensile modulus by 119%, while decrease the values of tensile strength and elongation at break by 50% and 43%, respectively, in the specimens containing 6-phr CS. In terms of the color appearances, the samples were lighter and yellower after the addition of CS, as evidenced by the noticeably increased L* and b* values, based on the CIE L*a*b* color space system. Furthermore, the investigation into the effects of thermal aging showed that the overall tensile properties for both curing systems were reduced, while varying degrees of color change were observed, with the pristine R-VNRL and RP-VNRL samples having more pronounced degradation/changes for both properties. In conclusion, the overall results suggested that CS had great potential to be applied as a bio-filler in R-VNRL and RP-VNRL composites to effectively promote the biodegradability, environmental friendliness, and resistance to thermal degradation of the composites.

## 1. Introduction

Rubber technologies, especially those related to natural rubber (NR), have been rapidly developed and extensively used in several applications, including flexible materials for radiation protection [1,2,3], medical and industrial latex gloves [4], polyethylene aerogel-coated natural rubber latex (NRL) foam for oil-water separation [5], a biodegradable proton exchanger in microbial fuel cells [6], and high-performance automotive tires [7]. This fast-growing utilization of NR has been mainly due to its superior properties compared to other synthetic rubber (SR), as NR is highly resilient and waterproof, while having a high stretch ratio and tensile/tear strength [8,9,10] that enables the materials to be used in applications requiring exceptional flexibility and strength.

While several curing systems have been introduced and used for the vulcanization of NR, ionizing radiation, especially gamma irradiation, is a common curing system used to produce radiation-vulcanized natural rubber latex (R-VNRL) products. The advantages of R-VNRL compared to a common sulfur-vulcanized natural rubber latex (S-VNRL) [11] are that the full vulcanization of natural rubber latex (NRL) can be achieved at normal temperature and pressure, leading to greatly reduced levels of cost, energy consumption, and pollution emitted during the curing processes [12]. In addition, R-VNRL products are considered much less hazardous than S-VNRL, because the latter normally require the use of toxic chemicals such as sulfur, -t-butyl-2-benzothiazolesulfenamide (TBBS), and Wingstay-L during rubber vulcanization and processing, as well as generating inevitable emissions of harmful by-products such as nitrosamines and nitrostables that may cause cancers and chemical allergies to humans, animals, and plants [13,14]. Therefore, the use of radiation, especially gamma rays, has gained great attention from researchers and product developers to develop a technology suitable for use on a larger industrial scale. Examples of recent developed R-VNRL products include X-ray shielding gloves based on nano-Bi_2_O_3_/R-VNRL composites [1], electromagnetic interference (EMI)-shielding materials from R-VNRL composites containing carbon nanotubes (CNT) and silk textile [14], and supercapacitor electrodes [15].

Recently, Ibrahim et al. reported a promising hybrid curing system using a combination of radiation and peroxidation. The results from their investigation indicated that the hybrid radiation and peroxide-vulcanized natural rubber latex (RP-VNRL) composites prepared using a combination of 0.1 phr tert-butyl hydroperoxide (*t*-BHPO) as a co-sensitizer and 6–10 kGy gamma irradiation exhibited improved overall mechanical properties compared to those of R-VNRL samples prepared using 12 kGy gamma irradiation (regarded as a control), as evidenced by the increases in the tensile modulus and tensile strength from 6 MPa and 20 MPa in the control to 11–13 MPa and 26–27 MPa in RP-VNRL, respectively, [16]. These studies not only reported some mechanical advantages from utilizing a hybrid curing system but also introduced improved methods to reduce the amounts of gamma doses and chemicals required for the full vulcanization of NRL, subsequently leading to both reduced cost and time to produce NRL products.

Although NR products could be regarded as biodegradable through the mechanism of microbial degradation, which makes them more environmentally friendly than other SR, rubber degradation is a time-consuming process, involving incubation periods amounting to weeks or months for any degradation or substantial weight loss to be observable [17,18]. This drawback was clearly emphasized by the report of Bras et al., which indicated that pristine NR lost just 19% of its original weight after being buried in soil for 4 weeks [19], while Jayathilaka et al. reported a weight loss of just 7–8% after soil burial for 14 weeks [20]. These two reports clearly implied that the disposal of NR wastes would take substantial amounts of time for complete biodegradation. Notably, differences in the biodegradation rates for the above examples could have been due to several factors, including the different types of microbes presented in the soil, different formulations and types of NR used for the fabrication of the specimens, and different biodegradation standards of testing [21].

To enhance the biodegradability of NR, several attempts have been made using the introduction of bio-fillers into NR composites to accelerate biodegradation processes. For example, Jacob et al. developed NR composites reinforced with varying sisal and oil palm fibers. Their investigation revealed that the percentage weight loss (*% weight loss*) after burial in the soil for 12 months was noticeably enhanced from ~1% in a pristine NR sample to ~11% in NR composites containing 25 phr of sisal and oil palm fibers [22]. Additionally, Supanakorn et al., showed that NR composites containing cellulose microfibers exhibited substantially enhanced biodegradability as the *% weight loss* after soil burial for 2 weeks increased from 3% in a pristine NR film to almost 70% in NR composites containing 50 wt.% cellulose microfibers [23]. These examples clearly indicate the advantages of incorporating appropriate bio-fillers into NR composites to enhance their rate of biodegradation.

In addition to the types of bio-fillers previously mentioned, chitosan (CS), which is a linear polysaccharide obtained from the hard outer skeleton of shellfish such as crab, shrimp, and lobster, is another interesting bio-filler that could be used for the enhancement of biodegradability in NR products [24]. In the past, CS was commonly added to different kinds of polymers, including NR, to enhance the antibacterial and antioxidant properties through bacteriostatic and bactericidal mechanisms [25] that resulted in improved useability and durability of the NR products [26,27,28]. However, thorough investigation of the role of CS as a biodegradation enhancer and of its effects on the physical and mechanical properties of NR composites, especially those prepared using radiation and hybrid radiation/peroxide curing systems, was still insufficient. Consequently, this lack of information has limited the full potential of CS utilization in rubber industries, which led to the need for further investigation. Some of the existing reports on the utilization of CS as a biodegradation enhancer in polymers were the development of poly(lactic acid)/CS film for use as food packaging and CS/polycaprolactone blends for the repair and regeneration of articular cartilage [29,30,31].

As mentioned, since CS were not fully investigated in its potential as biodegradation enhancers for NR, especially R-VNRL and RP-VNRL composites, the current work aimed to fulfill this gap by determining the biodegradability, physical (color), mechanical (tensile), chemical, and morphological properties of R-VNRL and RP-VNRL composites containing varying CS contents of 0, 2, 4, or 6 phr. The R-VNRL samples were prepared using gamma irradiation on NRL with a total absorbed dose of 15 kGy, while the RP-VNRL samples were prepared using the same chemicals and formulation as for R-VNRL, but, with the addition of 0.1 phr tert-butyl hydroperoxide (*t*-BHPO) as a co-sensitizer and a lower gamma irradiation dose of 10 kGy. The biodegradability of all samples was investigated by determining the percentage weight loss (*% weight loss)* of the samples after being buried in soil for 8 weeks, with the measurement of *% weight loss* carried out once a week. In addition, the effects of thermal aging on the CS/R-VNRL and CS/RP-VNRL composites were investigated by comparing the mechanical and physical properties of the composites before and after undergoing thermal aging. The outcomes of this work should not only support the development of CS as a biodegradation enhancer for R-VNRL and RP-VNRL composites but also increase the limited information and experimental data on the utilization of CS as a bio-filler in rubber industries that could enable production on larger scales.

## 2. Materials and Methods

### 2.1. Materials and Chemicals

**Figure 1:**
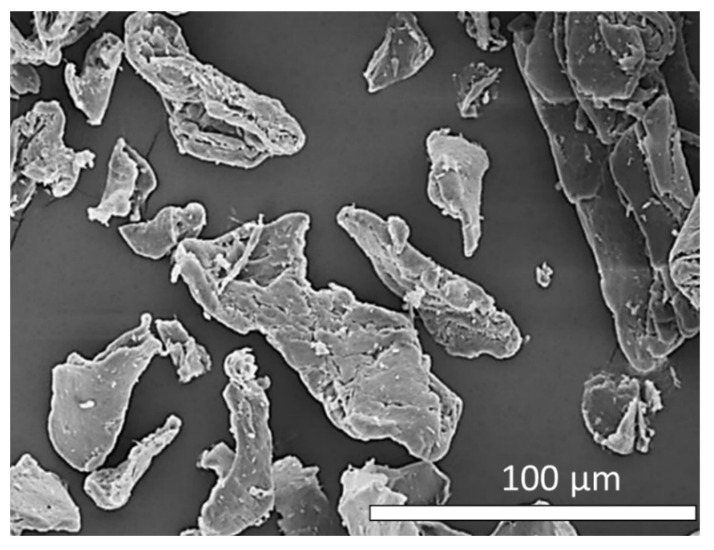
Micrograph image of chitosan (CS) used as a bio-filler in R-VNRL and RP-VNRL composites showing average particle size of 54 ± 5 µm. The image was taken at a magnification of ×500.

High-ammonia (HA) NRL used for the fabrication of testing specimens was supplied by the Office of Rubber Authority of Thailand (RAOT; Bangkok, Thailand) and its important characteristics are shown in Table 1. The formulations, including names of the chemicals, their roles, and their suppliers, for the fabrication of the R-VNRL and RP-VNRL composites are given in Table 2. The CS was obtained from shrimp shells (Marine Bio Resources Co., Ltd., Samut Sakorn, Thailand), with an average particle size (±standard deviation) of 54 ± 5 µm, a degree of deacetylation (%DD) of 82–87%, and an average molecular weight (*M*_w_) of 718 kDa. It should be noted that the average CS particle size was determined using micrograph images from scanning electron microscopy (SEM) (Figure 1) and the ImageJ software, version 1.50i (Madison, WI, USA).

### 2.2. Preparation of Chitosan (CS) Mixture

To improve the dispersion of CS in the NRL matrix, CS mixture was prepared using the formulations shown in Table 3. All chemicals were mixed and continuously stirred in a stainless-steel ball mill for 72 h before being kept in a closed container for further processing.

### 2.3. Preparation of CS/R-VNRL and CS/RP-VNRL Composites

NRL was continuously stirred using an automatic top stirrer/mixer at a rotating speed of 300 rpm for 30 min. Then, KOH was added to the NRL and the mixture was stirred for another 30 min. Next, either n-BA for R-VNRL or n-BA and *t*-BHPO for RP-VNRL was added and the stirring was continued for 40 min. After the stirring, the NRL mixture was transferred to a 5 L plastic container and irradiated with 15-kGy or 10-kGy gamma rays for R-VNRL or RP-VNRL samples, respectively. The irradiation procedure was performed at the Thailand Institute of Nuclear Technology (Nakhon Nayok, Thailand) using a ^60^Co source with a dose rate of 4.5 kGy/h. The gamma-irradiated NRL mixture was stored in a closed container at room temperature for 96 h before mixing with the prepared CS mixture using the automatic top stirrer/mixer for 2 h. It should be noted that the solid contents of CS added to NRL were varied (0, 2, 4, or 6 phr) with respect to the total solid rubber content of NRL and the maximum CS content used in this work was limited at 6 phr due to CS sinking rapidly to the bottom of the samples during the casting process at higher CS contents. The gamma doses of 10 kGy and 15 kGy used for the irradiation on NRL mixture were chosen based on the results of Ibrahim et al. [16] and Thumwong et al. [1] that gave the highest overall mechanical strengths for RP-VNRL and R-VNRL, respectively. To prepare the testing specimens, the CS/R-VNRL and CS/RP-VNRL mixtures were cast into 15 cm × 15 cm × 2 mm glass molds and left in open air for 3 days until the specimens became solid. Then, all testing specimens were peeled from the molds and kept in closed polyethylene bags for further measurement.

### 2.4. Thermal Aging on CS/R-VNRL and CS/RP-VNRL Composites

To determine the effects of thermal degradation on the mechanical and physical properties of the NRL composites, the specimens were placed in a hot-air oven at 100 °C for 24 h, following the procedure for the ASTM D5378-01a standard. After completion of the aging procedure, all specimens were tested for changes in mechanical, swelling, and color properties using the same procedures and standards testing as those for the non-aged specimens.

### 2.5. Characterization

#### 2.5.1. Biodegradability

**Figure 2:**
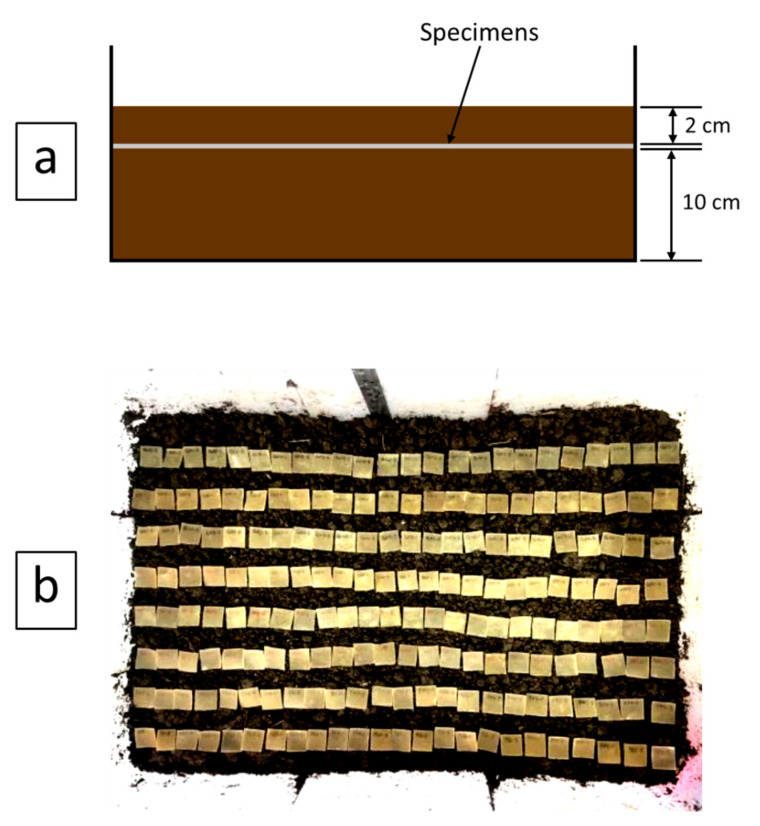
(**a**) Setup of biodegradability test (side view) and (**b**) actual arrangement of specimens on soil surface before being covered with another 2 cm layer of soil (top view).

The biodegradability of the CS/R-VNRL and CS/RP-VNRL composites was determined based on soil burial for a total of 8 weeks, following the testing procedure outlined by Bras et al. [19], where the soil was taken from the nearby surface layer and inert materials were removed from the soil. Then, the soil was ground and poured into a plastic container (50 cm × 80 cm × 30 cm) up to a thickness of 10 cm. It should be noted that the soil used in this work mostly composed of dark clay, with pH of 4.39 ± 0.01 (determined using procedure outline by Dai et al. [32]). The specimens with a dimension of 2 cm × 2 cm were placed systematically on the surface of the soil and another soil layer (2 cm thickness) was poured into the container such that all specimens were completely buried. Distilled water was sprayed twice daily on the soil surface to maintain the moisture content in the soil. At the end of each week, specimens were carefully taken out of the soil, washed with distilled water, oven-dried at 60 °C for 24 h, and stored in a desiccator for another 24 h. Next, the weight of each dried specimen was measured using a 4-digit scale (Practum224, Sartorius, Germany) and the percentage weight loss (*% weight loss*) was determined using Equation (1):

(1) $$\%weight\;loss=\frac{w_{o}-w_{1}}{w_{0}}\times 100\%$$

where w_0_ and w_1_ are the weights of specimen before and after soil burial, respectively. Notably that at least 3 specimens from each formulation and curing system were used to determine the *% weight loss*. The setup for the measurement of biodegradability is shown in Figure 2.

#### 2.5.2. Mechanical Properties

The tensile properties (tensile modulus, tensile strength, and elongation at break) of non-aged and thermal-aged specimens were determined using a universal testing machine (Autograph AG-I 5 kN, Shimadzu, Kyoto, Japan), according to ASTM D412-06 standard testing. The testing speed used for the measurement was 500 mm/min and each specimen was cut into a dumbbell shape (die C) prior to the measurement.

#### 2.5.3. Scanning Electron Microscopy (SEM) and Fourier-Transform Infrared Spectroscopy (FT-IR)

The morphological properties of the CS/R-VNRL and CS/RP-VNRL composites (both before and after being biodegraded) were determined using SEM with energy dispersive X-ray spectroscopy (SEM-EDX; Quanta 450 FEI: JSM-6610LV, Eindhoven, The Netherlands) at 10 kV accelerating voltage. Prior to the SEM measurement, the specimens were coated with gold using a sputter coater (Quorum SC7620: Mini Sputter Coater/Glow Discharge System, Lewes, UK) at a power voltage of 10 kV and a current of 10 mA for 120 s, to increase electrical conductivity of the surface [33,34].

Changes in the functional groups of the R-VNRL and RP-VNRL composites after the addition of CS were determined using FT-IR (Vertex 70, Bruker, San Jose, CA, USA), with wavenumbers in the range 500–4000 cm^−1^ [35].

#### 2.5.4. Swelling Behavior Studies

Swelling behaviors for non-aged and thermal-aged specimens were determined by measuring the percentage change in the weights (swelling ratio) of the specimens before and after soaking in toluene for at least 3 days or until constant weights were achieved. To obtain the swelling values, circular specimens with a diameter of 2 cm were soaked in toluene at room temperature, according to the procedures for the ASTM D471-06 standard. Initial weights before the immersion (*w*_1_) and weights after the immersion (*w*_2_) were measured using a 4-digit scale (Practum224, Sartorius, Germany) and the swelling ratio was then calculated using Equation (2):

(2) $$swelling\;ratio=\frac{w_{2}-w_{1}}{w_{1}}\times 100\%$$

#### 2.5.5. Surface Color Measurement

Color changes in the non-aged and thermal-aged specimens were determined using a colorimeter (HunterLab, Ultrascan Pro, Reston, VA, USA), following the CIE-L*a*b* colorimetric method, recommended by the Commission Internationale de l’Eclairage (CIE) [36]. The L*, a*, and b* parameters for each formulation, which represent the color positions of the specimen in a lightness axis, a blue-yellow axis, and a green-red axis, respectively, were determined and compared. The total color difference (Δ*E*) between the non-aged and thermal-aged specimens was calculated using Equation (3):

(3) $$\Delta E=\sqrt{{\left(L_{aged}^{*}-L_{0}^{*}\right)}^{2}+{\left(a_{aged}^{*}-a_{0}^{*}\right)}^{2}+{\left(b_{aged}^{*}-b_{0}^{*}\right)}^{2}}$$

where the parameters with the subscripts “_0_” and “_aged_” represent the color parameters obtained from the non-aged and thermal-aged specimens, respectively. A larger value of Δ*E* implies a higher degree of color difference between the two specimens, where values of Δ*E* in the ranges less than 1, 1–2, 2–10, 11–99, and 100 indicate the color differences between the two specimens were not perceptible to the human eye, by close observation, immediately, distinctly different, and exactly the opposite, respectively [37].

## 3. Results and Discussion

### 3.1. Determination of Functional Groups of CS/R-VNRL and CS/RP-VNRL Composites

**Figure 3:**
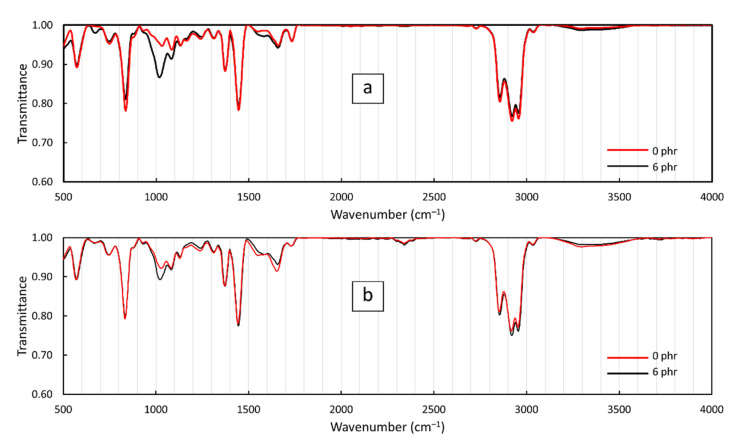
IR spectra of (**a**) pristine R-VNRL and 6-phr CS/R-VNRL composites and (**b**) pristine RP-VNRL and 6-phr CS/RP-VNRL composites.

The FT-IR spectra of the CS/R-VNRL and CS/RP-VNRL composites are shown in Figure 3a,b, respectively. For the pristine R-VNRL and RP-VNRL shown as red lines in Figure 3, dominant peaks were observed at 839 cm^−1^ (C=C bending), 1039 cm^−1^ (C–O stretching), 1085 cm^−1^ (C–O stretching), 1373 cm^−1^ (C–H bending), 1448 cm^−1^ (C–H bending), 1660 cm^−1^ (C=C stretching), 1773 cm^−1^ (C=O stretching), 2850–2960 cm^−1^ (C–H stretching), and 3215–3525 cm^−1^ (O–H stretching) [38]. The results of FT-IR for the pristine R-VNRL and RP-VNRL in this work agreed with the results from Ibrahim et al., which showed similar peak positions and band intensities for R-VNRL composites [39]. In particular, for the 6-phr CS/R-VNRL and 6-phr CS/RP-VNRL composites, peaks at 1039 and 1085 cm^−1^ were amplified compared to those of pristine samples. These enhanced band intensities of C–O stretching were mostly due to the added CS, which also had the dominant FT-IR peaks at around 1028 and 1066 cm^−1^ [40,41], resulting in noticeably higher band intensities at the peaks around 1039 and 1085 cm^−1^. These changes in the FT-IR results confirmed the presence of CS in the R-VNRL and RP-VNRL matrices.

### 3.2. Color Measurement of Non-Aged and Thermal-Aged CS/R-VNRL and CS/RP-VNRL Composites

The results of color determination on non-aged and thermal-aged CS/R-VNRL and CS/RP-VNRL composites are shown in Table 4, which indicates that the addition of CS as bio-fillers to R-VNRL and RP-VNRL composites resulted in the specimens being lighter and yellower, as evidenced by the increases in the values of L* (a lightness axis) and b* (a blue-yellow axis) from the ranges 42.23–48.79 and 6.18–6.34, respectively, in the pristine specimens to 58.56–65.48 and 8.24–12.88, respectively, in the specimens containing 6 phr of CS. The increases in these color parameters were due to the color indices of CS, which had relatively higher L* and b* values than those of R-VNRL and RP-VNRL (L* and b* for CS extracted from shrimp shells and prepared in an autoclave for 1–2.5 hr were reported to be in the ranges 75.65–83.21 and 10.01–12.56, respectively [42]). It is noteworthy that although the values of a* also increased after the addition of CS, which implied the specimens became slightly redder, their changes were much smaller than those for L* and b*, due to the comparable a* values of CS and R-VNRL/RP-VNRL (a* values of CS were reported to be in the range −0.18 to 1.80) [42]), resulting in less-pronounced changes observed in the green-red axis [36].

Table 4 also reported the values of L*, a*, and b* parameters for thermal-aged specimens. The results indicated that the thermal-aged specimens became darker and yellower after thermal degradation, as evidenced by the decrease in L* values (lightness axis) and the increase in b* (blue-yellow axis). Furthermore, the determination of Δ*E* revealed that changes in the colors of pristine R-VNRL and RP-VNRL specimens were more pronounced than those containing CS (the Δ*E* values for pristine R-VNRL and RP-VNRL specimens were greater than 10, implying their color differences were distinct), with the Δ*E* values decreasing with increasing CS contents. In particular, the Δ*E* values of the R-VNRL and RP-VNRL composites containing 6 phr of CS were 1.65 and 2.56, respectively, implying the color changes were only perceptible through close observation. The improved resistance to thermal degradation in terms of color appearance for the CS/R-VNRL and CS/RP-VNRL composites was probably due to slower physical degradation of CS compared to R-VNRL and RP-VNRL [43,44], consequently slowing down the effects of thermal degradation on the color appearances of composites.

### 3.3. Mechanical Properties of Non-Aged and Thermal-Aged CS/R-VNRL and CS/RP-VNRL Composites

**Figure 4:**
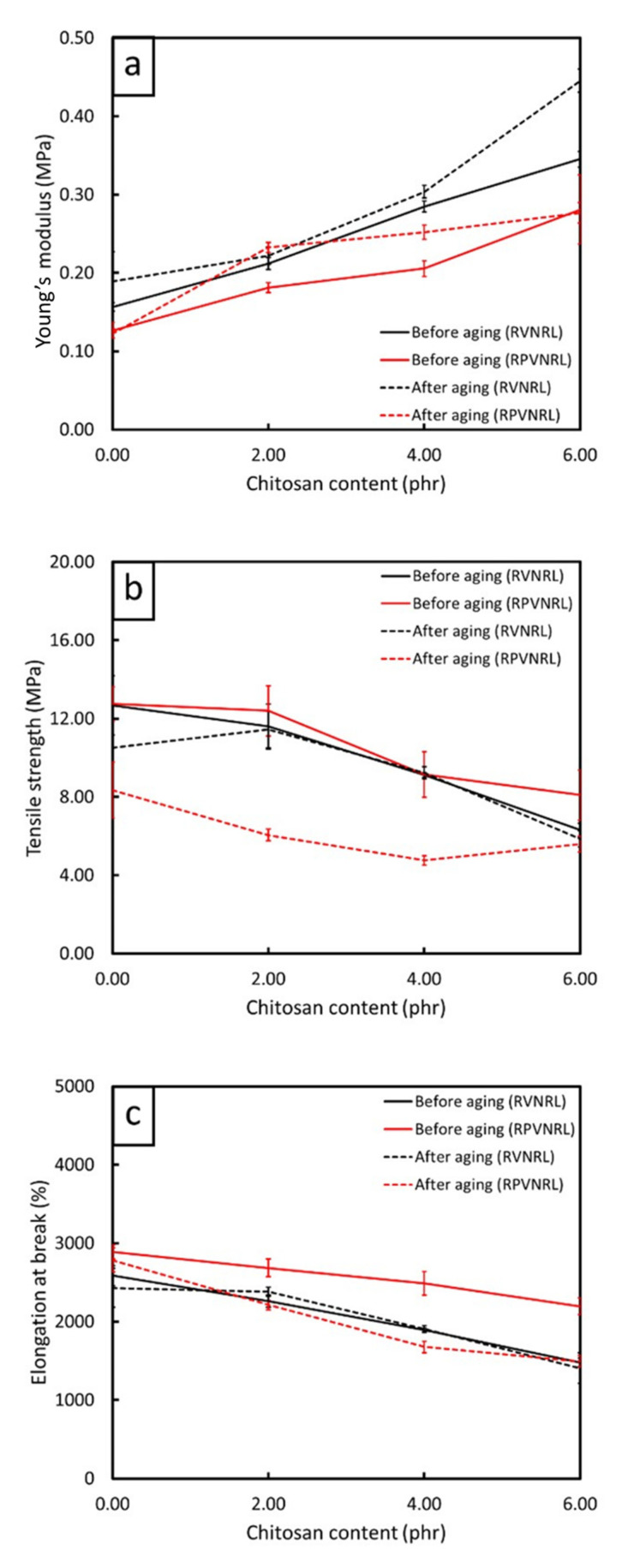
(**a**) Young’s modulus (tensile modulus at 500% elongation), (**b**) tensile strength, and (**c**) elongation at break of non-aged and thermal-aged CS/R-VNRL and CS/RP-VNRL composites, with varying CS contents of 0, 2, 4, or 6 phr. Error bars indicate ± standard deviation.

**Figure 5:**
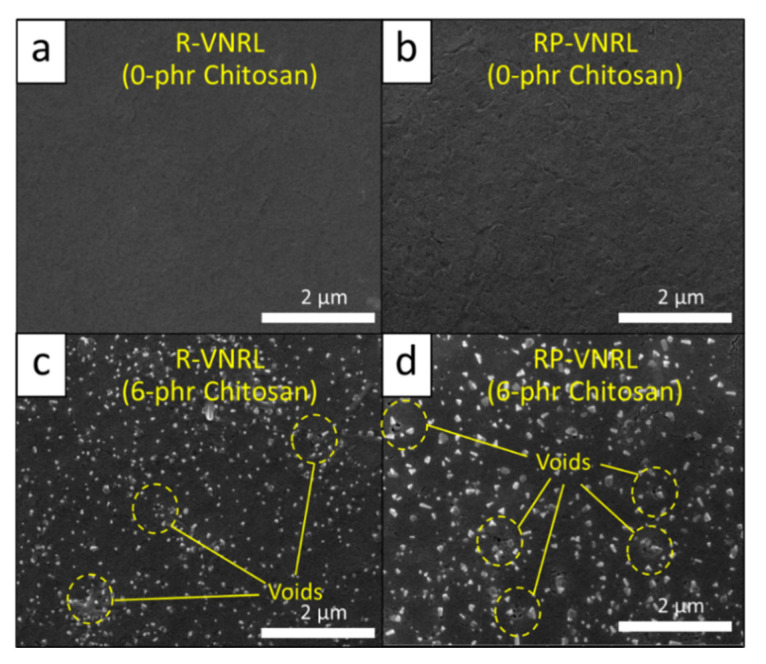
SEM images showing morphological properties of (**a**) pristine R-VNRL, (**b**) pristine RP-VNRL, (**c**) 6-phr CS/R-VNRL composites, and (**d**) 6-phr CS/RP-VNRL composites. All images are at a magnification of ×20,000.

**Figure 6:**
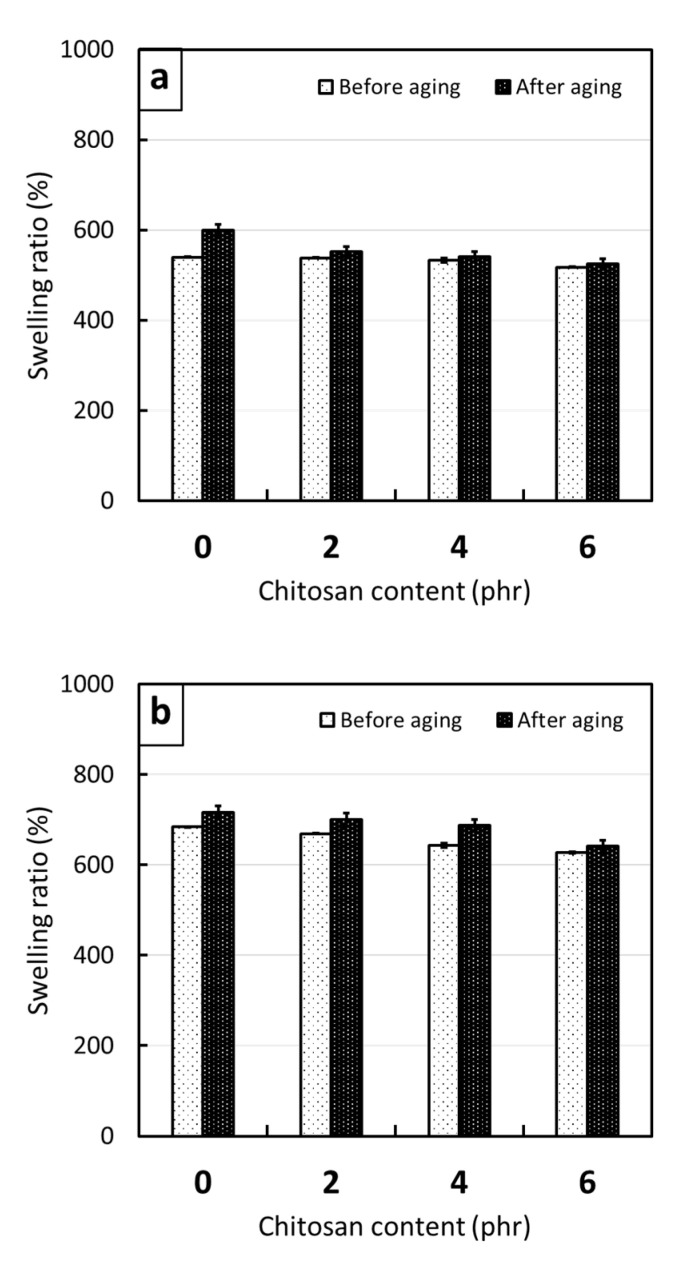
Percentage swelling (swelling ratio) of non-aged and thermal-aged (**a**) CS/R-VNRL composites and (**b**) CS/RP-VNRL composites, with varying CS contents of 0, 2, 4, or 6 phr. Error bars indicate ± standard deviation.

The tensile properties (tensile modulus, tensile strength, and elongation and break) of the non-aged and thermal-aged CS/R-VNRL and CS/RP-VNRL composites are shown in Figure 4 (the results of tensile modulus at 100, 200, 300, and 400% elongation are shown in Table S1, Supplementary Materials). For the non-aged specimens, the results indicated that the tensile modulus at 500% elongation tended to increase with increasing CS contents (up to 119% increase in the specimen containing 6-phr CS), while the tensile strength and elongation at break tended to decrease with increasing CS contents (decreases up to 50% and 43%, respectively, in the specimens containing 6-phr CS). The increase in tensile modulus after the addition of CS was mainly because of CS in limiting the movement of NR molecular chains that subsequently reduced the elasticity and increased the overall rigidity of the specimens [45]. On the other hand, the decreases in tensile strength and elongation at break after the addition of CS were mainly due to the poor interfacial compatibility between the CS particles and the NRL matrix, leading to visible voids and defects in the matrix (Figure 5c,d) [41]. Furthermore, irregular shapes of CS (Figure 1) could be another factor that reduced the overall strength of the composites as the ability to support the stress transferred from the NR matrix and the interaction between CS and the NR matrix were reduced [45]. It is noteworthy that the tensile modulus (elongation at break) of the CS/R-VNRL composites was higher (lower) than those of the CS/RP-VNRL composites with the same CS content. This was due to higher crosslink densities being formed in the CS/R-VNRL composites, as evidenced by lower swelling ratio values in the R-VNRL composites (Figure 6) (the swelling ratio value has a strong negative relationship with the material crosslink density [46]). The higher crosslink densities in the R-VNRL composites then limited the movement of NR molecular chains that subsequently increased the stiffness of the composites. It should be noted that the CS particles embedded in the NRL matrix (Figure 5) seemed to have smaller particle sizes than in the original CS. This reduction in particle size of CS was due to the use of a stainless-steel ball mill during the preparation of CS mixture that further reduced the sizes of CS particles (Section 2.2).

Figure 4 also shows the effects of thermal aging on the tensile properties of the CS/R-VNRL and CS/RP-VNRL composites, indicating that the thermal-aged specimens exhibited increased tensile modulus but decreased tensile strength and elongation at break after the specimens had undergone thermal degradation. These behaviors were due to two competing effects of thermal aging on the tensile properties of the composites. First, the thermal stress (heat) initiated the post-curing effect from the remaining chemicals, which further increased crosslink densities, rigidity, and, subsequently, the tensile modulus of the composites. Second, in contrast to crosslink formation from the post-curing effects, the applied heat commenced a chain-scission mechanism on the NR matrix, leading to oxidative degradation that fractured side-chain groups and the backbone of the NR molecular chains, consequently leading to the reduction of tensile strength and elongation at break of the materials that resulted in the materials becoming more brittle [34,47]. The presence of chain-scission after thermal aging was confirmed by the results of swelling ratio in Figure 6, which shows increased swelling ratios for thermal-aged specimens compared to the non-aged specimens with the same filler content and curing system. Notably, changes in swelling ratio between non-aged and thermal-aged pristine specimens were more pronounced than those containing CS.

In order to determine the abilities to resist thermal degradation of the composites, the aging coefficient (*S*) for each specimen was calculated using Equation (4):

(4) $$S=\frac{E_{\mathrm{aged}}\times TS_{\mathrm{aged}}}{E_{0}\times TS_{0}}$$

where *E*_aged_, *E*_0_, *TS*_aged_, and *TS*_0_ are the elongation at break of aged specimens, the elongation at break of non-aged specimens, the tensile strength of aged specimens, and the tensile strength of non-aged specimens, respectively [48]. The results of *S* obtained in this work are shown in Table 5, which indicated that CS/R-VNRL composites exhibited higher values of *S* than those of CS/RP-VNRL composites at the same CS content. The smaller values of *S* in RP-VNRL composites were mainly due to lesser crosslink densities (larger swelling ratios in Figure 6) in RP-VNRL than those in R-VNRL composites. It should be noted that the R-VNRL composites with the addition of CS (for all CS contents) had the *S* values greater than that of the pristine R-VNRL. This finding clearly indicated the enhanced resistance to thermal degradation of the CS/R-VNRL composites, mostly due to the known-property of CS in antioxidation [25].

### 3.4. Biodegradability

**Figure 7:**
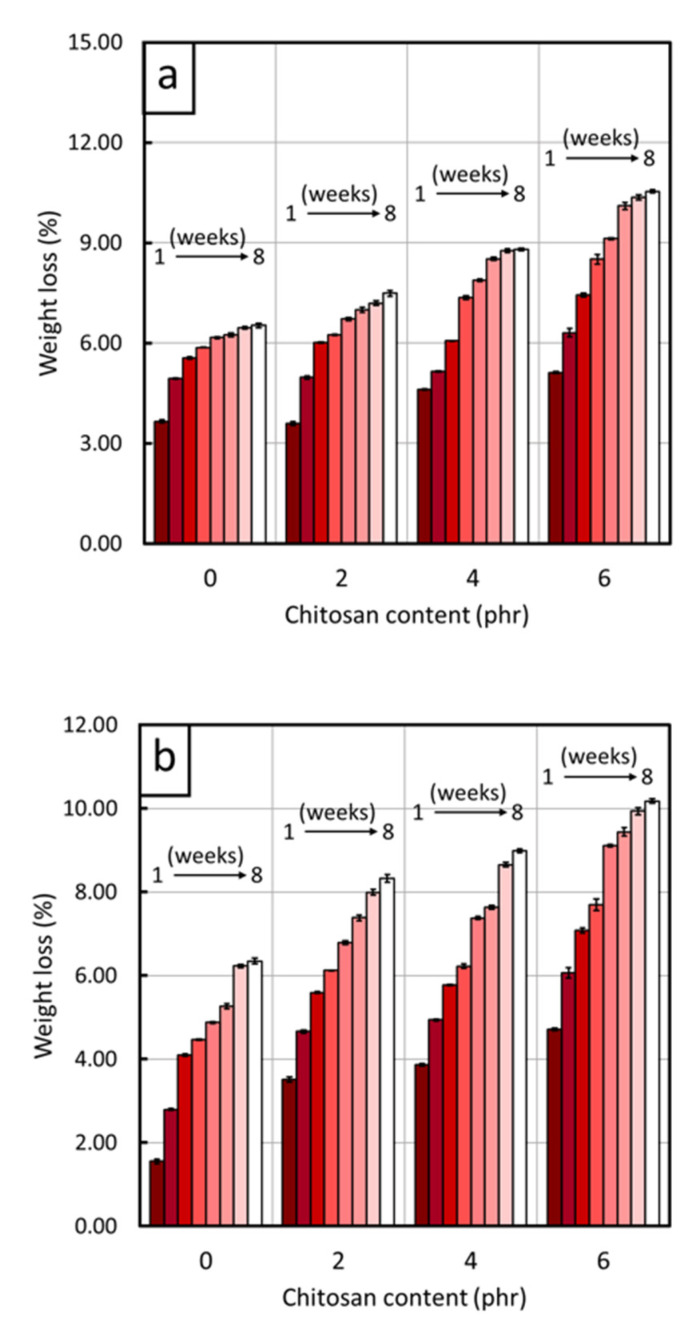
Percentage weight loss (*% weight loss*) of (**a**) CS/R-VNRL composites and (**b**) CS/RP-VNRL composites, with varying CS contents of 0, 2, 4, and 6 phr. The *% weight loss* was observed for a total of 8 weeks. Error bars indicate ± standard deviation.

**Figure 8:**
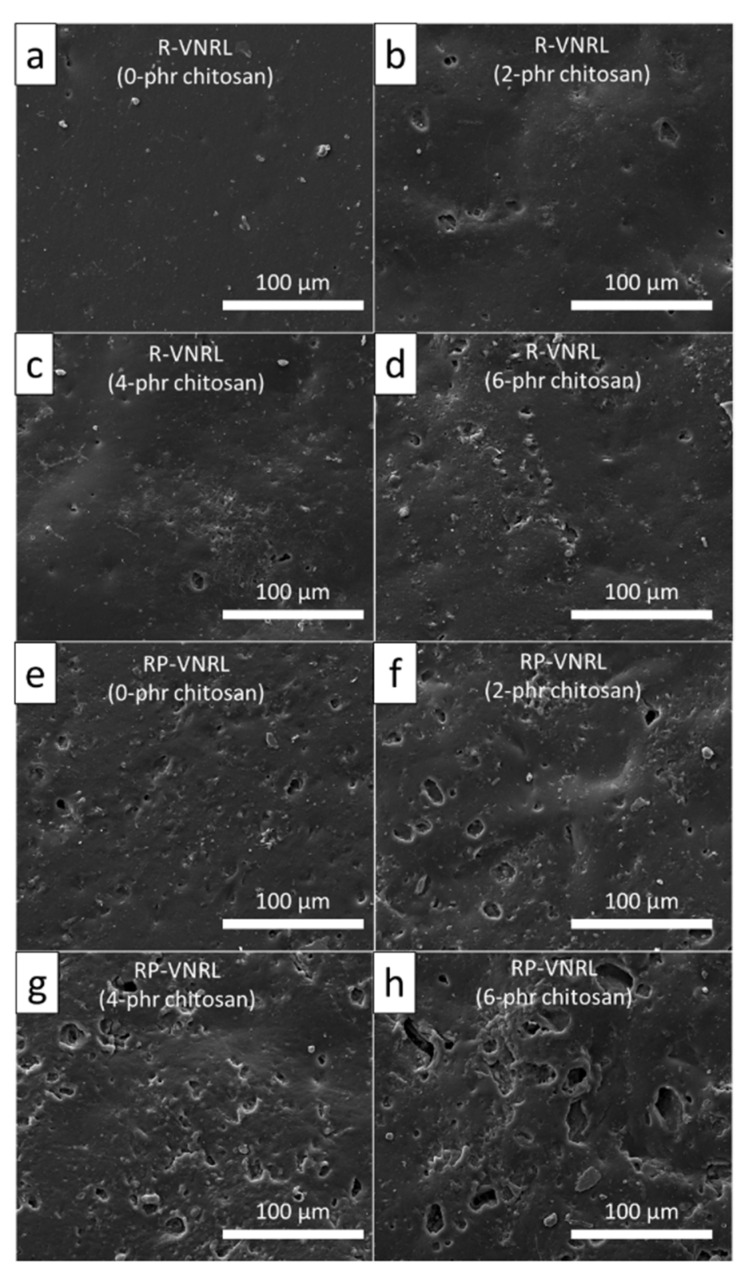
SEM images showing morphologies of (**a**) R-VNRL, (**b**) 2-phr CS/R-VNRL, (**c**) 4-phr CS/R-VNRL, (**d**) 8-phr CS/R-VNRL, (**e**) RP-VNRL, (**f**) 2-phr CS/RP-VNRL, (**g**) 4-phr CS/RP-VNRL, and (**h**) 6-phr CS/R-VNRL composites after the soil burial for 8 weeks.

The biodegradability, as represented by the *% weight loss* after soil burial for 8 weeks, of the CS/R-VNRL and CS/RP-VNRL composites is shown in Figure 7. The results indicated that the specimens exhibited varying degrees of biodegradability, with higher *% weight loss* in the specimens containing higher CS contents. For example, the *% weight loss* of pristine R-VNRL and RP-VNRL specimens after 8 weeks of soil burial were just 6.5 ± 0.1% and 6.4 ± 0.1%, respectively, while the R-VNRL and RP-VNRL composites containing 6 phr of CS exhibited *% weight loss* values of 10.5 ± 0.1% and 10.2 ± 0.1%, respectively, indicating noticeably enhancement in their biodegradability of 61.5% and 59.4%, respectively. These results were due to the faster biodegradation of CS by microorganisms compared to those of NR, as reported by Nakashima et al., where CS film exhibited *% weight loss* values of 98.9% and 99.1% after being buried in red clay and paddy soil, respectively, for just 1.5 months [49]. This fast degradation of CS resulted in the increased void formation and the loss of integrity of the NR matrix; so that the NR matrix could be broken down into smaller particles by microorganisms in shorter periods of time [19,50]. The micrograph images of all biodegraded specimens are shown in Figure 8, which revealed that specimens containing CS, especially at 6 phr (Figure 8d,h), clearly had more and larger voids in the R-VNRL and RP-VNRL matrices (average void sizes of 15.1 ± 2.2 µm and 26.2 ± 3.4 µm, respectively) compared to the pristine R-VNRL and RP-VNRL (average void sizes of 2.5 ± 0.7 µm and 9.7 ± 1.2 µm, respectively). These findings positively supported the biodegradation results, which indicated higher *% weight loss* in the specimens with high CS contents. The accelerated *% weight loss* and biodegradability of the R-VNRL and RP-VNRL composites with the addition of CS implied that CS effectively acted as a bio-filler and enhanced the biodegradability of the composites, which could reduce substantially the amount of time needed for waste disposal.

## 4. Conclusions

This work determined the chemical, physical, mechanical, morphological, and biodegradability properties of R-VNRL and RP-VNRL composites containing varying CS contents (0, 2, 4, or 6 phr). The FT-IR spectra indicated that CS was successfully embedded into the NRL matrix, which resulted in the color of the specimens being lighter and yellower compared to the pristine specimens. The results also indicated that the addition of CS enhanced the tensile modulus at 500% elongation by 119% and *% weight loss* (biodegradability) by approximately 60% after 8-week soil burial but reduced the tensile strength and elongation at break of the composites by 50% and 43%, respectively. The investigation into the effects of thermal aging on the swelling, mechanical, and physical properties of the composites revealed that varying degrees of changes could be observed, namely, the color of thermal-aged specimens became darker and yellower (with less pronounced changes found in the composites containing CS). Furthermore, the thermal aging on the specimens led to the increases in the tensile modulus and swelling ratio, while reducing tensile strength and elongation at break of the composites. The overall outcomes of this work suggested great potential for the utilization of CS as a bio-filler in R-VNRL and RP-VNRL composites to enhance the biodegradability and environmental friendliness of the composites and to expand the already-known advantages of CS as an antibacterial and antioxidant enhancer in rubber industries such that CS could be utilized to its fullest potential.

## Figures and Tables

**Table 1:** Important characteristics of high-ammonia natural rubber latex (NRL) used in this work.

Characteristic	Value
Total solid content (%)	60.9
Dry rubber content (%)	60.1
Ammonia content (on total weight; %)	0.7
pH	10.5
Viscosity (cP)	75.3

**Table 2:** Components of natural rubber latex (NRL) used during gamma irradiation for the fabrication of radiation-vulcanized natural rubber latex (R-VNRL) and hybrid radiation and peroxide-vulcanized natural rubber latex (RP-VNRL) composites and their roles and suppliers.

Component	Content (phr *)	Role	Supplier
HA-NRL	100	Matrix	RAOT (Bangkok, Thailand)
10% Potassium hydroxide (KOH)	0.25	Stabilizer	Gammaco Co., Ltd. (Bangkok, Thailand)
100% n-Butyl acrylate (n-BA)	5	Sensitizer	VIV Interchem Co., Ltd. (Bangkok, Thailand)
100% tert-Butyl hydroperoxide (*t*-BHPO) **	0.10	Co-sensitizer	Richest Group (Shanghai, China)
Distilled water	30	Solvent	Kasetsart University (Bangkok, Thailand)

* phr: parts per hundred part of rubber (by weight); ** t-BHPO was only used for the fabrication of RP-VNRL composites.

**Table 3:** Components for preparation of chitosan (CS) mixture and their suppliers.

Component	Content (%wt)	Supplier
Chitosan (CS)	10	Marine Bio Resources Co., Ltd. (Samutsakorn, Thailand)
1% Ammonia (NH_3_)	10	Gammaco Co., Ltd. (Bangkok, Thailand)
Vultamol	1	RAOT (Bangkok, Thailand)
Bentonite clay	1	RAOT (Bangkok, Thailand)
Distilled water	78	Kasetsart University (Bangkok, Thailand)

**Table 4:** Color indices of non-aged and thermal-aged CS/R-VNRL and CS/RP-VNRL composites with varying CS contents of 0, 2, 4, or 6 phr.

Sample	Chitosan Content (phr)	Before Aging			After Aging			Δ*E*
		L*	a*	b*	L*	a*	b*	
R-VNRL	0	48.79	−0.56	6.34	45.89	−0.06	16.02	10.11
	2	65.48	−0.66	9.04	61.31	0.42	13.59	6.27
	4	59.57	−0.17	8.24	60.63	0.38	9.26	1.57
	6	58.56	0.31	9.11	57.31	0.17	10.19	1.65
RP-VNRL	0	42.23	−0.32	6.18	45.93	0.33	16.77	11.24
	2	62.23	−0.36	9.47	61.79	0.15	13.92	4.50
	4	65.13	0.31	10.40	61.03	0.45	12.98	4.85
	6	59.99	0.6	12.88	58.76	0.62	15.12	2.56

**Table 5:** Aging coefficients (*S*) of non-aged and thermal-aged CS/R-VNRL and CS/RP-VNRL composites with varying CS contents of 0, 2, 4, or 6 phr.

Sample	Chitosan Content (phr)	Aging Coefficient (*S*)
R-VNRL	0	0.77
	2	1.05
	4	1.05
	6	0.87
RP-VNRL	0	0.62
	2	0.39
	4	0.32
	6	0.43

## Data Availability

The data presented in this study are available upon request from the corresponding author.

## Supplementary Materials

The following are available online at https://www.mdpi.com/article/10.3390/polym13223940/s1, Table S1: Stress at 100, 200, 300, and 400% elongation of non-aged and thermal-aged CS/R-VNRL and CS/RP-VNRL composites with varying CS contents of 0, 2, 4, or 6 phr.

## Nomenclature

ASTM	American Society for Testing and Materials
Bi_2_O_3_	Bismuth oxide
CNT	Carbon nanotubes
CS	Chitosan
%DD	Degree of deacetylation
Δ*E*	Total color difference
FT-IR	Fourier-transform infrared spectroscopy
HA	High ammonia
KOH	Potassium hydroxide
*M*_W_	Molecular weight
n-BA	n-Butyl acrylate
NH_3_	Ammonia
NRL	Natural rubber latex
% weight loss	Percentage weight loss
phr	Parts per hundred part of rubber (by weight)
RAOT	Rubber Authority of Thailand
RP-VNRL	Radiation and peroxide-vulcanized natural rubber latex
RV-NRL	Radiation-vulcanized natural rubber latex
SEM	Scanning electron microscope
SR	Synthetic rubber
S-VNRL	Sulfur-vulcanized natural rubber latex
*t*-BHPO	Tert-butyl hydroperoxide
