# Supplementary material for: Roles of Chitosan as Bio-Fillers in Radiation-Vulcanized Natural Rubber Latex and Hybrid Radiation and Peroxide-Vulcanized Natural Rubber Latex: Physical/Mechanical Properties under Thermal Aging and Biodegradability
Source: Polymers (Basel). 2021 Nov 15;13(22):3940. doi: 10.3390/polym13223940 (PMC8618650; doi:10.3390/polym13223940)
Supplement: Supplementary file 1 [file polymers-13-03940-s001.zip › polymers-1449312-supplementary.pdf]

Supplementary Materials

Table S1. Stress at 100, 200, 300, and 400% elongation of non-aged and thermal-aged CS/R-VNRL and CS/RP-VNRL composites with varying CS contents of 0, 2, 4, or 6 phr.

Sample	Chitosan content (phr)	Stress at 100% elongation (MPa)		Stress at 200% elongation (MPa)		Stress at 300% elongation (MPa)		Stress at 400% elongation (MPa)	
		Before aging	After aging	Before aging	After aging	Before aging	After aging	Before aging	After aging
R-VNRL	0	0.03	0.03	0.07	0.07	0.10	0.11	0.13	0.15
	2	0.04	0.05	0.10	0.11	0.14	0.15	0.18	0.18
	4	0.05	0.05	0.11	0.12	0.17	0.19	0.22	0.24
	6	0.06	0.07	0.15	0.18	0.23	0.29	0.29	0.37
RP-VNRL	0	0.03	0.03	0.06	0.07	0.09	0.09	0.11	0.11
	2	0.04	0.05	0.07	0.09	0.11	0.15	0.15	0.20
	4	0.04	0.05	0.08	0.11	0.14	0.16	0.17	0.21
	6	0.06	0.06	0.13	0.13	0.18	0.19	0.23	0.23
